# Healing and survivorship: what makes a difference?

**DOI:** 10.3747/co.v15i4.288

**Published:** 2008-08

**Authors:** H.D. Braude, N. Macdonald, M. Chasen

**Affiliations:** * Biomedical Ethics Unit, McGill University, Montreal, QC.; † Cancer, Nutrition and Rehabilitation Programme, McGill University, Montreal, QC.

**Keywords:** Cancer, healing, narrative, rehabilitation, survivorship, social relationships

## Abstract

Literature demonstrating the importance of social relationships for cancer survivorship is accumulating. Building on that literature, the term “Healing Ties” refers to the scientific and popular factors supporting the idea that relationships and community are essential for healing. However, difficulties arise in assessing the effect of social support for survivorship.

The current paper reviews the role in survivorship of social support, with respect to the explanatory model provided by neuro-oncology and psycho-neuro-immunology. Taking cognizance of the importance of social relationships, the model of cancer rehabilitation aims, through its interdisciplinary framework, to restore a sense of well-being and to facilitate healing by optimizing the capability for full social relationships and engagement with the world.

## 1. INTRODUCTION

In recent decades, a paradigm shift has occurred in oncology, from treatment of disease to treatment of the whole person. In his influential *The Structure of Scientific Revolutions* 1, Thomas Kuhn argued that scientific theories do not evolve from the straightforward accumulation of facts, but from a set of changing historical and contextual circumstances. Medical practice is closely aligned with this concept, because medicine is a combination of science, art, and the humanities 2.

## 2. HEALING TIES

In her recent cultural history of mind–body medicine, the Harvard-based historian Anne Harrington refers to the constellation of scientific and popular factors informing particular theories as “narratives.” Narratives are templates, she writes 3:

They provide us with tropes and plotlines that help us understand that larger import of specific stories we hear, read, or see in action. They also help us construct specific stories of our own—including ones about our own experience—that others can recognize and affirm. We learn these narrative templates from our own culture, not in the way we might formally learn the rules of grammar in school, but in the way we might unconsciously learn the rules of grammar at home—by being exposed to multiple examples of living stories that rely on them.

One particular narrative that Harrington describes as “Healing Ties” refers to the simple but revolutionary idea that social relationships and community are essential for the prevention of illness and the promotion of healing and well-being.

The concept of social stress as a causal determinant for (ill) health was advanced through the pioneering studies of notable figures in social epidemiology such as John Cassell, Len Symes, and Michael Marmot. Marmot’s work—made famous through his Whitehall studies looking at British civil servants—emphasizes the importance of the social inequality gradient as an independent indicator of health and well-being 4.

Another related field, psychobiology, examines the pathways through which psychosocial factors stimulate biologic systems by central nervous system activation of autonomic, neuroendocrine, and immunologic responses 5. In a classic study that applied those ideas to cancer, Vernon Riley showed that rates of tumour growth and quickened mortality were associated with a stressful environment 6. In mice with the Bittner tumour virus, a group raised in harsh surroundings showed an accelerated tumour onset and course as compared with a group maintained, apparently, as mice like to be maintained. This basic science model accords with studies examining the effects of social relationships for human cancer survivorship.

In his 1978 review of research in psycho-oncology, Bernard Fox hypothesized about the interconnected biologic and psychological pathways that should be studied to determine the influence of states of mind on the promotion of cancer and its subsequent progress 7:

Cancer is a multi-step process; whatever the random mutation and other biological initiators of the cancer process, its further production and spread will depend in part on homeostatic controls that can be influenced by psychological factors through neurohormonal and immunological pathways. This hypothesis is valuable in focusing the attention of researchers on the patient, stimulating studies on the following: (1) the influence of specific states of mind on etiology (promotion) and prognosis of certain cancers, (2) the effect of psychological therapy on duration of survival of patients, and (3) the biological pathways that medicate the posited effects of states of mind.

In the decades since Fox’s review was published, a number of studies (but not all) have demonstrated the association between social support and increased survival for patients with various cancers a.

The conflicting evidence about the importance of psychological interventions for cancer survivorship and healing is also exemplified by three seemingly identical randomized controlled trials concerning the effects of group therapy on life expectancy for women suffering with late-stage (metastatic) breast cancer. The initial study in 1989 by the psychiatrist David Spiegel and colleagues reported that a cohort of women in support groups appeared to live, on average, twice as long as a control cohort not in such groups 15; more recent studies seem convincingly to controvert that finding 16,17. Admittedly, these studies deal with the very narrow context of group therapy and not the broader one of social support as provided by family and community, but Harrington uses Spiegel’s example to question the validity of the template of Healing Ties.

The difficulty in assessing the effect of social support for survivorship exists in defining the terms of research in narrow methodologic language, in evaluating quantitatively that which is essentially numinous, and then in determining the causative role of the defined idea in a particular disease. Yet despite the complexity of the design process, sophisticated techniques are currently being developed, particularly in relation to the collateral health effects of social networks 18.

Although the weight of evidence suggests that social support increases survival for people living with cancer, it is certain that social support can help to reduce distress and suffering. The emphasis on assessing longevity, important as it is, might actually diminish the perceived importance of social relationships for experiential aspects of being ill and caring for ill people, an essential element of relationship-centered medicine 19.

## 3. WHOLE-PERSON CARE

If the shift toward whole-person care is a recent phenomenon in modern Western medicine, the insights upon which it draws are ancient. Plato is often justifiably invoked as the father of medical holism. Thus, in a well-known dialogue in the *Phaedrus* (270c), Plato compares the art of medicine with rhetoric. Here, Socrates says that whereas the task of medicine is to define the nature of the body, the task of rhetoric is to define the nature of the soul. Moreover, one cannot know the nature of the soul without knowing the nature of the whole. To which Phaedrus responds that Hippocrates the Asclepiad says that even the nature of the body can be understood only as a whole. Medicine, like hermeneutic philosophy b relates part to whole, and whole to part.

Commenting on this passage, the philosopher Hans-Georg Gadamer in his essays on the enigma of health notes that “the nature of the whole includes and involves the entire life situation of the patient, and even of the physician” 20. It is in the manner that medicine relates the disease to the person and to the larger community that the ultimate validity for the importance of social relationships in the health care context needs to be sought, rather than in the results of any one particular study. As noted by Plato, there is a parallel between the structure of the body—the subject of medicine—and the structure of the psyche, the self (or in Platonic terms, the soul).

## 4. CANCER REHABILITATION

To elucidate the foregoing idea in contemporary terms, consider the example of cancer rehabilitation, a movement that exemplifies the shifting emphasis from diagnosis of disease to symptom specificity or whole-patient care. Perhaps the key concept for cancer rehabilitation is that of interdisciplinarity. Rehabilitation requires an interdisciplinary team approach because of the variety of potential problems that patients may face during the course of their illness 21. For this reason, the cancer rehabilitation team that we are involved with includes people trained in the disciplines of medicine, nursing, dietetics, physiotherapy, psychology, social work, occupational therapy, and medical ethics. One consequence of this interdisciplinarity is the acknowledgment that rehabilitation and healing can occur only when treatment incorporates as many relevant approaches and stories as have a bearing on the well-being of the person—including, of course, those of the patient and the patient’s family. The emphasis on social relationships is an important aspect of the approach espoused in cancer rehabilitation. Not only are social relationships important for particular interventions, but the aim of cancer rehabilitation is to restore a sense of well-being of self by optimizing the capability for full social relationships and engagement with the world.

## 5. SUMMARY: STORIES AS THE HEALING MATRIX OF SOCIAL RELATIONSHIPS

As indicated earlier, the effects of social relationships are not, however, simply social, but biological. Consider cachexia. There are both biologic reasons (the association of chronic inflammation with tumour progress and symptoms) and social imperatives to support a comprehensive care model from the time of diagnosis for cancer patients 22. The effects of interdisciplinary care directly affect the constellation of physiologic factors that are associated with cancer. At a conceptual level, strengthening social relationships allows healing and survivorship to occur because it provides support for an ailing organism to self-organize. The constellation of pathologic factors is matched by the constellation of care that crystallizes in the narrative template of Healing Ties.

Just as social stress provokes biologic weakening of an organism, social relations provide the opportunity for the whole person to flourish through the optimization of the physiologic functions upon which the human self is based. It is therefore not surprising that there is also evidence to suggest that the act of storytelling itself has positive effects on health and well-being. The social psychologist James Pennebaker has demonstrated in well-replicated studies the immunologic benefits that follow disclosure of traumatic events 23. Stories are not just stories, but an essential part of the feedback loop between the body and the self, the biologic and cultural matrix for the healing effects of social relationships. These stories may not come forward in the usual sequential trajectories of cancer care—a situation that isolates biochemotherapy from other components of cancer rehabilitation. They do emerge in the course of including patients and their families as partners in a community of care—a central tenet of cancer rehabilitation.

## Footnotes

a See, for example, Barraclough *et al.,* 1992 8; Goodkin *et al.,* 1986 9; Goodwin *et al.*, 1987 10; Graham *et al.,* 2002 11; Neale *et al.,* 1986 12; Priestman *et al.,* 1985 13; and Waxler–Morrison *et al.,* 1991 14.

b “Hermeneutics” refers to the methodologic principles of textual interpretation. Hermeneutic philosophy analyses the philosophical foundations of interpretative methodologies. Hans–Georg Gadamer, one of whose essays is quoted in the next paragraph, is the most notable contemporary exponent of this philosophical method.
